# Pharmacologic Comparison of High-Dose Hesperetin and Quercetin on MDCK II Cell Viability, Tight Junction Integrity, and Cell Shape

**DOI:** 10.3390/antiox12040952

**Published:** 2023-04-18

**Authors:** Mio Nakashima, Natsuko Goda, Takeshi Tenno, Ayaka Kotake, Yuko Inotsume, Minako Amaya, Hidekazu Hiroaki

**Affiliations:** 1 Laboratory of Structural Molecular Pharmacology, Graduate School of Pharmaceutical Sciences, Nagoya University, Furocho, Chikusa-ku, Nagoya 464-8601, Aichi, Japan; 2 BeCerllBar, LLC, Business Incubation Building, Nagoya University, Furocho, Chikusa ku, Nagoya 464-8601, Aichi, Japan; 3 Cosmetics Research Department, Nicca Chemical Co., Ltd., Fukui 910-8670, Fukui, Japan; 4 Center for One Medicine Innovative Translational Research, Gifu University Institute for Advanced Study, Yanagito, Gifu 501-1112, Gifu, Japan

**Keywords:** tight junction integrity, absorption enhancer, dynamic equilibrium of tight junction, claudin-ZO-1 interaction, TGFβ pathway, MEK pathway

## Abstract

The modulation of tight junction (TJ) integrity with small molecules is important for drug delivery. High-dose baicalin (BLI), baicalein (BLE), quercetin (QUE), and hesperetin (HST) have been shown to open TJs in Madin-Darby canine kidney (MDCK) II cells, but the mechanisms for HST and QUE remain unclear. In this study, we compared the effects of HST and QUE on cell proliferation, morphological changes, and TJ integrity. HST and QUE were found to have opposing effects on the MDCK II cell viability, promotion, and suppression, respectively. Only QUE, but not HST, induced a morphological change in MDCK II into a slenderer cell shape. Both HST and QUE downregulated the subcellular localization of claudin (CLD)-2. However, only QUE, but not HST, downregulated CLD-2 expression. Conversely, only HST was shown to directly bind to the first PDZ domain of ZO-1, a key molecule to promote TJ biogenesis. The TGFβ pathway partially contributed to the HST-induced cell proliferation, since SB431541 ameliorated the effect. In contrast, the MEK pathway was not involved by both the flavonoids, since U0126 did not revert their TJ-opening effect. The results offer insight for using HST or QUE as naturally occurring absorption enhancers through the paracellular route.

## 1. Introduction

The tight junction (TJ) is the apical-most intercellular adhesion complex found in epithelial and endothelial cells [1,2,3]. TJs are a proteinous complexes, containing integral membrane proteins, such as occludin (OCLN) and claudins (CLDs), as well as cytosolic scaffold proteins, such as zonula occludens (ZO)-1 (and its close paralog ZO-2), which connect these membranous components to the actin cytoskeleton [4,5,6,7]. As the key physicochemical function of TJ is to limit the free translocation of solvents, solutes, and cells through the paracellular pathway, TJs play a pivotal role in the barrier function of the paracellular transport pathway. TJs have a barrier function in the paracellular pathway, since they restrict the free translocation of solvents, solutes, and cells across the epithelial cell layers. Hence, TJs are particularly important for the digestive organs, respiratory tract, skin, and blood vessels. In a pharmacological context, the barrier function of TJs sometimes hampers the absorption of medium-sized drugs, including peptides and oligonucleotides, across paracellular pathways. Accordingly, the reversible modifiers of TJ integrity are expected to act as drug absorption enhancers.

ZO-1 and ZO-2 are essential components that promote TJ biogenesis and maintain TJ integrity [7,8]. These scaffold proteins harbor N-terminal three copies of the postsynaptic density 95 (PSD-95)/discs large/ZO-1 (PDZ) domains, followed by the Src-homology 3 domain and a guanylate kinase domain [4,9,10]. Among these three PDZ domains of ZO-1/2, the first PDZ domain of ZO-1 (ZO-1 (PDZ1)) acts as the molecular interface to bind the C-terminal PDZ-binding motifs (PBMs) of CLDs [11]. This specific interaction has been shown to be indispensable for TJ formation and maintenance [7]. For example, the cells of ZO-1 and ZO-2 double knockdown decreased some (but not all) CLD localizations in the TJ area, with an abnormal accumulation of apical actin [12]. Similarly, ZO-1 knockout/ZO-2 knockdown resulted in abnormal and immature organization of CLDs in the TJ compartment [13]. The fact that the point mutations in the PBMs of CLDs exhibited TJ-defective phenotypes partly supports the relevance of the ZO-1(PDZ1)–CLD interaction [14]. The number of examples of molecules that directly bind to ZO-1(PDZ1)) with TJ-opening activities is also increasing [15,16,17,18,19].

To discover milder and safer TJ modifiers, we focused on TJ-modulating flavonoids. Flavonoids are generally believed to be beneficial with regards to consumption from foods, medical herbs, and traditional medicines, and this is the result of their antioxidant and anti-inflammatory properties [20]. In this study, we focused on the other pharmacological activity of flavonoids, rather than their antioxidative activity. In our previous studies, baicalin (BLI) and baicalein (BLE) showed specific binding to ZO-1(PDZ1) using nuclear magnetic resonance (NMR) methods, with mild TJ-opening activity in two epithelial cells: Madin–Darby canine kidney (MDCK) II cell and Caco-2 cell [18]. We also reported that a high-dose administration of hesperetin (HST) and quercetin (QUE) against MDCK II cells resulted in reduced subcellular localization of CLD-2 to the TJ compartment and decreased TJ integrity [21]. Note that there are no previous investigations into the TJ-opening activity of HST. In contrast, the reported TJ opening of QUE seems controversial, since many other groups have reported barrier-enhancing and/or barrier-protecting activity of QUE for epithelial cells [22]. Nevertheless, nephrotoxicity was also reported by an overdose administration of QUE [23]. Thus, elucidation of the pharmacological mechanism of the TJ opening of HST and QUE may facilitate the development of a safer TJ-opening modulator from natural sources, especially from citrus fruits.

In this study, we compared the effects of flavonoids on cell viability, cell shape morphology, TJ modulating activity, and mechanisms of TJ opening with regards to the effects of flavonoids on MDCK II cells. We also assessed the direct interaction of ZO-1(PDZ1) with either HST or QUE through NMR titration experiments using ^15^N-labelled ZO-1(PDZ1). Finally, we discussed the application potential of HST and QUE as ‘safer’ drug absorption enhancers.

## 2. Materials and Methods

### 2.1. Materials

HST (>96% pure), QUE (practical grade), SB431542, and U0126 were purchased from FUJIFILM Wako Pure Chemical Corporation (Osaka, Japan). All compounds used for NMR and cell experiments were dissolved into *d*_6_-dimethylsulfoxide (DMSO) as a 100 mM solution and stored at −20 °C until needed.

The rabbit anti-CLD-2 antibody and anti-OCLN antibody were obtained from Sigma-Aldrich (St. Louis, MO, USA). The rabbit anti-ZO-1 antibody was obtained from Invitrogen (Carlsbad, CA, USA). Mouse anti-β-actin antibody was purchased from Wako. Rhodamine–phalloidin was purchased from Cytoskeleton, Inc. (Denver, CO, USA). For immunofluorescence microscopy, the anti-rabbit immunoglobulin G (IgG) and the F(ab’)2 fragment-Cy3 antibody were obtained from Sigma-Aldrich. For Western blotting analysis, anti-rabbit and mouse IgG horseradish peroxidase (HRP) conjugates were acquired from Promega (Madison, WI, USA).

### 2.2. Cell Culture and Morphology Analysis

The culture of MDCK II cells, a kind gift from Mikio Furuse (National Institute for Physiological Sciences, Okazaki, Aichi, Japan), and their morphology analysis was performed according to our previous reports [18,21]. For this process, Dulbecco’s modified Eagle’s medium (DMEM) supplemented with 10% fetal bovine serum (FBS; Biosera, Ringmer, UK), 1% penicillin/streptomycin (Gibco, NY, USA, or Wako Pure Chemical Co.), and six-well, 35-mm plate (AGC Techno Glass Co., Shizuoka, Japan) were used, and 30 × 10^4^ cells per well were plated. To observe the flavonoids’ and the inhibitors’ effect, the culture medium was changed to a medium containing 100 μM of HST and QUE, or 5, 10, and 20 µM of SB431542 and U0126, supplemented with a final concentration of 0.1% of *d*_6_-DMSO, after twenty-four hours after plating. Cells treated by 0.1% *d*_6_-DMSO were used as controls. The cells were analyzed using immunofluorescent microscopy, Western blotting, real-time polymerase chain reaction (PCR), a WST-8 assay, and cell morphology analysis (differential interference contrast (DIC) images) after 48 h of exposure to the compounds.

The cell viability after compound exposure was monitored by WST-8 assay. 1.5 × 10^4^ cells per well were plated on a 96-well, 7-mm plate (AGC Techno Glass Co.). The culture medium was changed to a medium containing 100 μM of HST and QUE, or 5, 10, and 20 µM of SB431542 and U0126, with a final concentration of 0.1% of *d*_6_-DMSO added after one day after plating. After two days of treatment with the compounds, cell viability was measured using a Cell Counting Kit-8 (DOJINDO, Kumamoto, Japan). An EnSpire plate reader (Perkin-Elmer Japan, Kanagawa, Japan), excited at 450 nm, was used.

To obtain the morphological parameters of the cell shape, DIC-images were digitalized and subjected to ImageJ software (National Institute of Health, Rockville Pike, Bethesda, MD, USA). All parameters were normalized against their corresponding values from the control cells.

### 2.3. Immunofluorescence Microscopy

After fixation of the cells with cold 1 × phosphate-buffered saline, containing 4% paraformaldehyde, the cells were incubated with primary antibodies (anti-CLD-2, OCLN, ZO-1, and actin) for 24 h at 4 °C. The cells were then incubated with secondary antibodies for 1 h. The fluorescence images were obtained using fluorescence microscopy (IX-71, Olympus, Tokyo, Japan; scale bar: 20 µm) equipped with a color charge-coupled device camera DP-70 (Olympus, Shinjuku, Tokyo, Japan). For CLD-2 and actin immunostaining, brightness (+85%) and contrast (+10%) in Figures 2, 3, and 6 were modified for clarity. The original figures are found in the Supplementary Figures.

### 2.4. Western Blotting

Western blot analysis was performed according to our previous reports [18,21]. The cells were rinsed, and crude proteins were extracted with 100 µL of the buffer, containing sodium dodecyl sulfate (SDS). Mild sonication, using a Bioruptor (BM Equipment Co., Tokyo, Japan), for 5 min (duty cycle 50%), was used for this process. Each crude protein sample was analyzed by SDS–polyacrylamide gel electrophoresis. The samples were then electroblotted to the poly(vinylidene fluoride) (PVDF) membrane (ATTO, Tokyo, Japan) and blocked with 3% skim milk for overnight with the primary antibodies. The membrane was washed four times and treated with corresponding secondary antibodies. Detection of the corresponding proteins was achieved using a Chemi-Lumi One Super solution (Nacalai Tesque, Kyoto, Japan) and the LAS-3000 analyzer (Fuji Film, Tokyo, Japan). For quantification of the results from the Western blotting, the experiments were repeated at least three times. The number of the repeats was indicated in the figure legends.

### 2.5. Real-Time PCR

The RNA was purified using the RNeasy Plus Mini Kit (QIAGEN, Tokyo, Japan), based on the manufacturer’s instructions. Then, cDNA was prepared using the ReverTra^®^ Ace qPCR RT Master Mix (Toyobo Co., Osaka, Japan), based on the manufacturer’s instructions. Quantitative real-time PCR was performed using the LightCycler^®^ System (Roche Diagnostics, Tokyo, Japan). The primer sequences are as follows: *GAPDH* (GenBank accession number: NM_001003142) (Fw 5′-CAACTCC-CTCAAGATTGTCAGCAA-3′ and Rev 5′-CATGGATGACTTTGGCTAGAG-GA-3′), *CLD-2* (GenBank accession number: NM_001003089) (Fw 5′-CGCTCCGACTACTATGACTCCT-3′ and Rev 5′-GGCCTTGGAG-AGCCTCTAGT-3′), *OCLN* (GenBank accession number: NM_001003195) (Fw 5′-CTGGAGCAGGACCACTATGAGA -3′ and Rev 5′-CTCCTCCAGCTCGTCACAC-3′), and *TJP1* (GenBank accession number: NM_001003140) (Fw 5′-GGAG-ATTCCGGGGT-CTTCG-3′ and Rev 5′-CTGGCTGAGCTGACAAATCCTC-3′). An aliquot of 1 μL of template cDNA with 0.6 μL of forward and reverse primers was mixed with 10 μL of THUNDERBIRD^®^ SYBR^®^ qPCR Mix (TOYOBO, Osaka, Japan) and adjusted to a total solution volume to 20 μL.

### 2.6. Protein Expression and Purification

The expression and purification of the mouse ZO-1(PDZ1) (residues 18–110) has been previously described with a slight modification [24]. In brief, ZO-1(PDZ1) was expressed as the glutathione-S-transferase (GST)-tagged form by Escherichia coli BL21 (*DE3*). For the NMR sample, 1 L M9 minimal media with ^15^N-ammonium chloride as the sole nitrogen source was used [24]. The fusion protein was captured using GST-accept (Nacalai Tesque). To obtain ^15^N-labeled ZO-1(PDZ1), the fusion protein was digested “on-column” using PreScission™ Protease. Finally, the sample was further purified by size-exclusion chromatography using a Superdex 75 column (Cytiva, Tokyo, Japan).

### 2.7. NMR Titration Experiments

To assess the direct interaction between ZO-1(PDZ1) and the flavonoids, NMR titration experiments were employed, in which a series of 2D ^1^H-^15^N heteronuclear single quantum coherence (HSQC) spectra at 25 °C were analyzed. The sample was dissolved in 5% D_2_O–95% H_2_O containing 20 mM of MES buffer (pH 5.9). In the titration, up to two molar equivalences of HST and QUE were added to 0.1 mM ^15^N-ZO-1(PDZ1), and the normalized chemical shift changes Δδ_normalized_ in the 1H-^15^N HSQC spectra upon ligand titration were analyzed as follows (Equation (1)),

Δδ_normalized_ = {Δδ(^1^H)^2^ + [Δδ(^15^N)/5]^2^}^1/2^ (1)

where Δδ(^1^H) and Δδ(^15^N) are the chemical shift changes in the amide proton and amide nitrogen, respectively [25]. The results were visualized using the molecular graphics tool PyMOL program (The PyMOL Molecular Graphics System, Version 2.0, Schrödinger, LLC., Broadway, New York, USA) onto the ribbon representation of the ZO-1(PDZ1) structure (the Protein Data Bank (PDB) ID = 2H3M). Each threshold value was calculated using the method developed by Schumann et al. [26].

### 2.8. Molecular Docking Based on NMR Chemical Shift Perturbations

Structural models of the ZO1-HST and ZO1-QUE complexes were calculated with the NMR structure of ZO-1(PDZ1) (PDB ID of 2RRM) [16] using HADDOCK software (HADDOCK2.4, Utrecht University, Heidelberglaan, Utrecht, The Netherlands), which derives the docking model, fulfilling the chemical shift perturbations (CSPs) [27,28]. We used the first model registered in 2RRM for the docking experiment. The data of CSPs were used as the restraints to generate the NMR-based docking model, according to the user manual. Thus, the residues of ZO-1(PDZ1) that showed marked CSPs were defined as the binding sites of ZO-1(PDZ1). The ZO1-HST structure with the lowest Z score was selected and displayed using PyMOL. The coordinates of the flavonoids were obtained from the webserver (https://molview.org (accessed on 1 December 2021)) and converted into pdb format. The other details are described in Supplementary Information.

### 2.9. Statistical Analysis

Statistical analyses were performed by one-way ANOVA followed by a Tukey–Kramer test. A difference of *p* < 0.05 was considered significant. All values are expressed as means with their standard errors of mean.

## 3. Results

### 3.1. Cell Viability of HST or QUE

**Figure 1:**
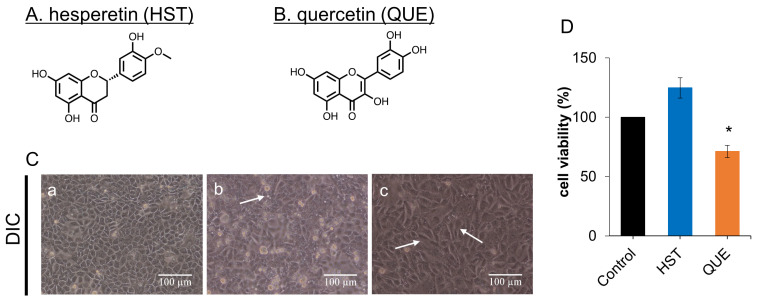
Chemical structures of (**A**) hesperetin (HST) and (**B**) quercetin (QUE). (**C**) Morphological change in Madin-Darby canine kidney (MDCK) II cells induced by flavonoids. Bright-field differential interference contrasts (DIC) images with corresponding flavonoids that are arrayed. Cells were exposed to flavonoids at a concentration of 100 μM for 48 h. Arrows show the changes. (**a**) Control (DMSO), (**b**) HST, and (**c**) QUE. Scale bar = 100 µm. (**D**) Effects of flavonoids on cell viability in MDCK II cells. Cells were treated with flavonoids at a concentration of 100 μM for 48 h. Error bars indicate standard deviations. Turkey-Kramer multiple comparison tests were applied as statistical analyses. Difference from the value of the control cells, * *p* < 0.05. HST, QUE: *n* = 4.

We assessed the conditions (concentrations and time points) of the HST and QUE treatments that did not damage the MDCK II cells severely. The MDCK II cells began to form TJs approximately 24 h after seeding. We chose this time point for treatment with flavonoids and examined the effects on the proliferation of MDCK II cells. As shown in Figure 1D, the cells treated with HST showed an increased viability of up to 130%, whereas those treated with QUE showed a decreased viability of 60% compared to the control. This drop in cell viability after exposure to 100 µM if QUE raises the concern of toxicity of a high-dose QUE treatment, although QUE is considered a beneficial flavonoid.

### 3.2. Changes in Cell Morphology Induced by QUE

We then assessed the morphological changes in the MDCK II cells exposed to 100 µM of HST and QUE under a bright-field phase contrast microscope. In our previous studies, we reported pharmacological effect of several flavonoids against MDCK II cells at the concentration between 50 and 100 µM, and moderate effect were observed after 48 to 96 h of exposure [18,21]. Thus, in this study, we also chose 100 µM and 48 h as the fixed experimental conditions. It is worth noting that the concentration is below the 50% cytotoxicity concentration of QUE (2500 µM) and HST (2900 µM) against MDCK cells [29]. When the cells were exposed to HST, the cell monolayer seemed normal and healthy in terms of shape and size. However, we found many white granular spots (white spots) (Figure 1C panel b, arrow, Supplementary Figure S2). Similar white spots, such as oil droplets, were repeatedly observed in our previous study [21]. Moreover, when the cells were exposed to high concentrations (200 µM) of HST, several holes in the cell monolayer formed due to unknown cellular stress of HST.

By contrast, the cells treated with QUE showed significant morphological changes compared to the typical cobblestone-like morphology of untreated MDCK II cells, which exhibited an elongated and slenderized cell shape similar to a fibroblast-like appearance (Figure 1C panel c, arrows). It should be noted that we also reported similar morphological changes and a fibroblast-like phenotype of MDCK II cells when the cells were exposed to a high dose of BLE [18].

We quantitatively analyzed these morphological changes using QUE by estimating the length of the transverse and longitudinal axes of the cells using the cell areas from microscopic images. We found that the cells elongated by 180% in the long-axis direction. When the cells were treated with QUE for 96 h, the elongation reached 220%, with 130% elongation of the short axis. As a result, the relative cell area became more than 300% (Supplementary Figure S1). Surprisingly, these morphological changes were irreversible. The cells did not recover and were re-transformed into a native shape even after 48 h of the removal of QUE (Supplementary Figure S1). These changes seemed specific to QUE and were not observed in the cells exposed to rutin, a 3-rutinoside of QUE [21].

**Figure 2:**
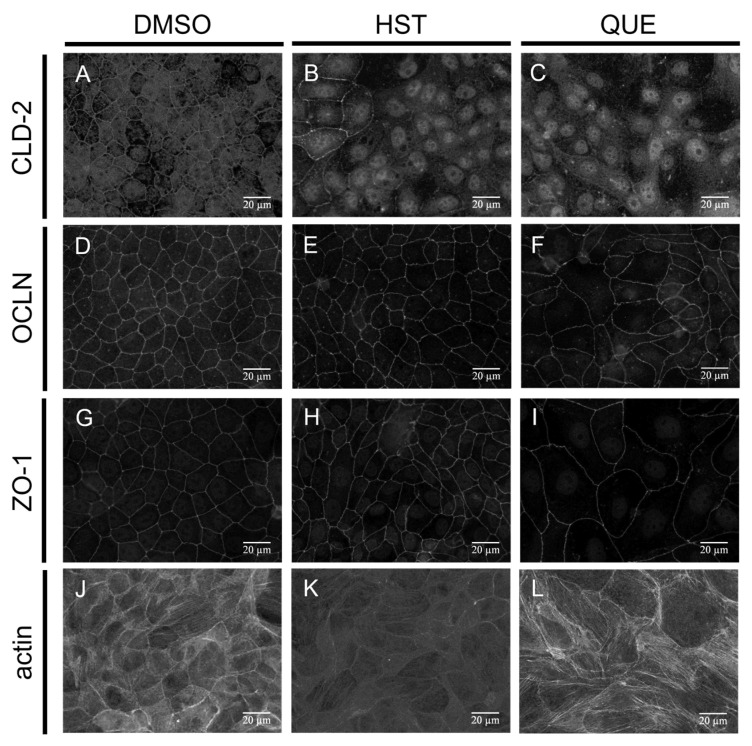
Changes on TJ integrity of MDCK II cells induced by flavonoids. Immuno-fluorescence staining of CLD-2, ZO-1, OCLN, and actin were applied to the MDCK II cells exposed to flavonoids at a concentration of 100 μM for 48 h. (**A**,**D**,**G**,**J**) control (DMSO); (**B**,**E**,**H**,**K**) HST; (**C**,**F**,**I**,**L**) QUE. Scale bar = 20 µM. Brightness of images was modified to 160%.

We hypothesized that the QUE-induced morphological changes could be one of the important steps of the “partial” epithelial–mesenchymal transition (EMT), which we previously observed in BLE treatment [18]. If so, then this observation is somewhat controversial in the many reported QUE activities, as QUE can prevent EMT in many cancer cells [30,31]. Thus, we assessed whether the observed morphological changes were signs of partial EMT. For this purpose, we assessed the subcellular distributions of CLD-2, OCLN, ZO-1, and actin cytoskeleton, as the disintegration of the intercellular cell–cell junctions is one of the critical checkpoints of EMT. The localization of TJ-related proteins (CLD-2, ZO-1, and OCLN) was observed using immunofluorescence microscopy. As shown in Figure 2, the significant morphological changes in the MDCK II cells exposed to QUE were readily more visible using fluorescent immunological microscopy with paraformaldehyde fixed cells than using bright-field microscopy (Figure 2F,I). In addition, the amount of CLD-2, which was localized at the cell–cell interaction membrane compartment, decreased (to be discussed later). However, the localization of ZO-1 and OCLN was not affected by QUE treatment, suggesting that EMT did not occur (Figure 2F,I). We also assessed the amount and direction of cortical actin bundles. We could not find any significant changes in actin compared to the control (Figure 2L), suggesting that the regulatory pathway of the actin cytoskeleton might not be a direct target of QUE.

The same experiment was performed for HST, and similar to QUE, the membrane localization of OCLN, ZO-1, and actin fibers was not affected. Therefore, although we observed cell slenderizing induced by QUE, neither QUE nor HST induced partial EMT and rearrangement of the actin cytoskeleton of MDCK II cells.

### 3.3. TJ Reduction Activity of HST or QUE

**Figure 3:**
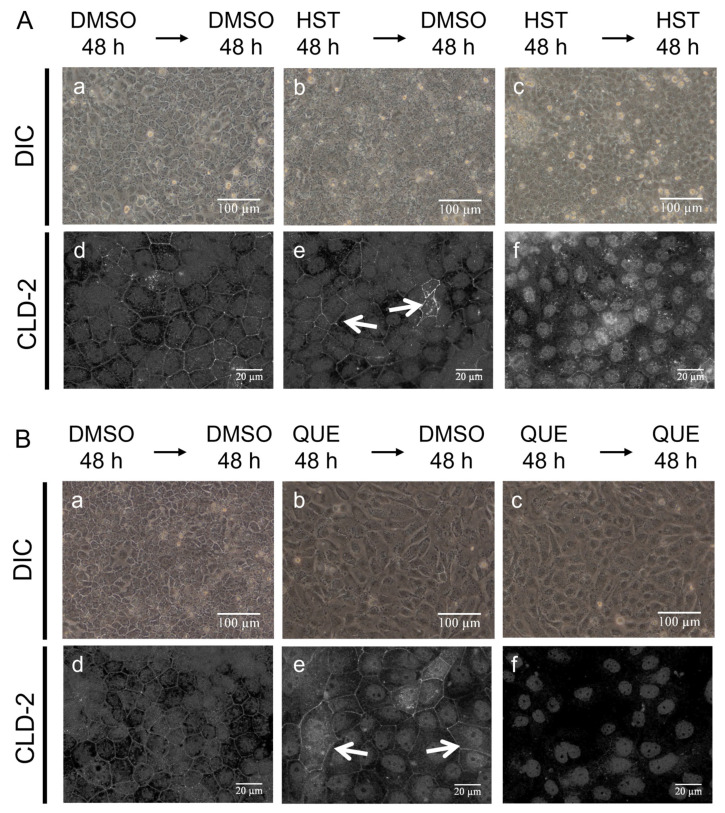
Partially irreversible changes of morphology and TJ integrity of MDCK II cells induced by (**A**) HST or (**B**) QUE. DIC images (**a**–**c**) and immunofluorescence-stained images of CLD-2 (**d**–**f**) of MDCK II cells are shown. The cells were exposed to HST or QUE at a concentration of 100 μM for 48 h and then treated with HST, QUE, or DMSO at a concentration of 100 μM for 48 h after washing with the medium. (**a**,**d**) Control (DMSO–DMSO), (**b**,**e**) flavonoid–DMSO, and (**c**,**f**) flavonoid–flavonoid. Scale bar = 100 µm (**a**–**c**), 20 µm (**d**–**f**). For immunofluorescence staining, brightness was modified to 160%. Arrows indicate CLD-2 localized at regenerated tight junctions.

During the assessment of the possibility of partial EMT by QUE or HST through immunofluorescence microscopy, we succeeded in reproducing the TJ-opening activity of QUE and HST (Figure 2B,C), which we previously reported [21]. The amount of CLD-2 in the TJ compartment significantly decreased after 48 h of exposure to 100 μM QUE or HST. As CLD-2 is considered the most abundant CLD in MDCK II cells [32], we supposed that these changes were related to the decreased integrity of TJ. We then examined the reversibility of the flavonoid-induced TJ-opening of MDCK II cells and continued observing the recovery of TJ-accumulated CLD-2 48 h after QUE was washed out. The amount of TJ-accumulated CLD-2 was partially restored after 48 h in the medium without QUE after QUE treatment (Figure 3B, panel e). The same experiment was also performed for the HST. In the bright-field observation of living MDCK cells, the white spots on the cell monolayer surface induced by HST vanished due to HST removal. Accordingly, the TJ-accumulated CLD-2 was restored after a 48-h culture in the medium without HST after HST treatment (Figure 3A panel e). Therefore, the effects of QUE or HST on CLD-2 attenuation in the TJ compartment were partially reversible, whereas the effect of QUE on cell morphology seemed irreversible.

**Figure 4:**
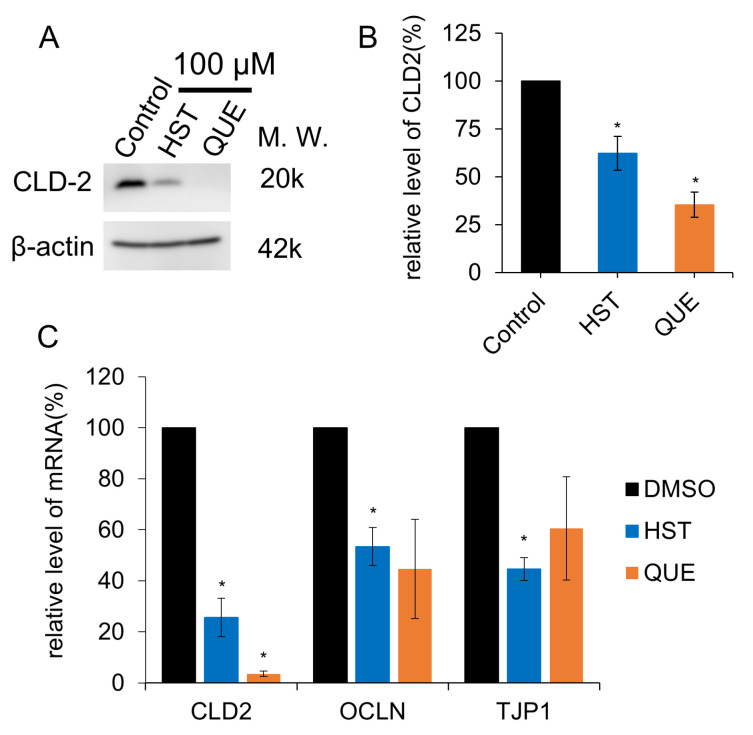
Semi-quantitative analyses of protein or mRNA levels of CLD-2, OCLN, and ZO-1 (TJP1) after 100 µM flavonoid exposure for 48 h in MDCK II cells. (**A**) Western blotting analysis of CLD-2 expression in cell lysates from the control (DMSO) and 100 µM flavonoid-treated MDCK II cells. (**B**) Quantitative analysis with densitometry of 100 µM flavonoid-treated MDCK II cells. HST, QUE: *n* = 8. (**C**) RT-qPCR analysis of CLD-2, OCLN, and TJP1 expressions in cell lysates from the control (DMSO) and 100 µM flavonoid-treated MDCK II cells. Error bars indicate standard deviations. Statistical analyses were performed using the Turkey–Kramer multiple comparison tests. Difference from the value of the control cells, * *p* < 0.05. HST, QUE: *n* = 3.

We then examined the protein expression level of CLD-2 in cells treated with QUE and HST using Western blotting. Figure 4A shows the results of the amount of CLD-2 extracted from cells treated with 100 μM HST or QUE after 48 h of exposure. Figure 4B is a bar graph of the protein level of CLD-2 treated with 100 μM HST or QUE normalized to the untreated cells. The CLD-2 protein level decreased to 65% and 35% compared to the controls with HST and QUE treatment, respectively. These changes were dose-dependent, and treatment with 10 μM of HST or QUE did not induce observable changes in the CLD-2 levels in our preliminary experiments. Accordingly, we examined the reversibility of CLD-2 reduction by analyzing the cells that were first exposed to either HST or QUE and then incubating with HST- or QUE-free medium for 48 h. Western blot analysis showed that the removal of HST or QUE from the medium could restore CLD-2 protein levels.

As shown previously, we also analyzed the effects of HST and QUE on the other TJ-related proteins, ZO-1 and OCLN. ZO-1 is a protein that serves as a scaffold during TJ formation, and its mislocalization from TJ decreases TJ integrity. After exposure to either HST or QUE, we observed the subcellular localization of ZO-1 in the MDCK II cells (Figure 2H,I). Similarly, the subcellular localization of OCLN was also monitored (Figure 2E,F). However, the amount of ZO-1 and OCLN localized in the lateral membrane of MDCK II cells was not affected, regardless of the decrease in CLD-2. This suggests that the decrease in the subcellular localization of CLD-2 is independent from those of OCLN and ZO-1 upon HST or QUE treatment.

In addition, we examined the changes in CLD-2 mRNA levels upon HST and QUE treatment using quantitative PCR. The CLD-2 mRNA level was significantly decreased by HST or QUE (Figure 4C). QUE was more potent than HST in terms of CLD-2 transcription suppression. Surprisingly, although the protein levels of OCLN and ZO-1 were not drastically changed, their mRNA levels were suppressed by HST and QUE.

### 3.4. NMR Evidence of Direct Binding of HST but Not QUE to ZO-1(PDZ1)

We previously succeeded in determining the solution structure of mouse ZO-1(PDZ1) using solution NMR experiments found that the C-terminal peptide of CLD-3 and phosphatidylinositol phosphate competitively bound to the canonical peptide binding site of ZO-1(PDZ1) [16]. In a recent study, ZO-1(PDZ1) was directly inhibited by glycyrrhizin, which prolongs TJ-opening induced by deoxycholate in the Caco-2 cell monolayer [33]. These results suggest that the direct binding of a small molecule ligand to the CLD binding site of ZO-1(PDZ1) may inhibit the physiologically important interaction between ZO-1(PDZ1) and CLDs, which may result in the malformation of TJ or at least disturb TJ integrity in epithelial cells. According to this hypothesis, the flavonoids BLI and BLE directly bound to the CLD binding site of ZO-1(PDZ1) and reduced TJ integrity [18]. Thus, we again examined whether HST and QUE directly bound to ZO-1(PDZ1) using the solution NMR technique. In the HSQC spectra of ^15^N-labeled ZO-1(PDZ1), small but certain chemical shift perturbations (CSP) were observed with the addition of two equivalents of HST (Supplementary Figure S5A). The major residues with a marked CSP of the amide signals were found to surround the canonical peptide-binding pocket of ZO-1(PDZ1) (Supplementary Figure S5A panels b,c). The binding mode of HST to ZO-1(PDZ1) was predicted by the NMR-based HADDOCK approach, and the putative complex structure seemed reasonable (Supplementary Figure S5A panel d,e). HADDOCK is one of the docking simulation software for both protein–protein and protein–small ligdocking, which is specialized to utilize the data from NMR titration experiments with the user-friendly interface [28]. As a result, HADDOCK succeeded in predicting the binding site of HST as same as the canonical CLD binding site on ZO-1(PDZ1) [16]. Thus, we hypothesized that the direct inhibition of HST to ZO-1(PDZ1) is one of the mechanisms that promotes TJ-opening.

By contrast, although the chemical structure of QUE is similar to HST, no remarkable CSP of ZO-1(PDZ1) upon QUE titration was observed (Supplementary Figure S5B panel b,c) in 2 molecular equivalents. Thus, we performed HADDOCK simulation without NMR-based restraints. As a result, the binding sites of QUE were not converged into the canonical ligand binding pocket (Supplementary Figure S5B panel d). Nevertheless, interestingly, one of the best five models fitted the equivalent binding site of HST (panel e). The difference between the two flavonoids is the relative orientation between A and B rings of flavanone (HST) and flavonol (QUE), respectively, may explain the difference of ZO-1 binding. In detail, the A and B rings of QUE are kept planar, whereas the A and B rings of HST are skewed (Supplementary Figure S5B panel f). In addition, it should be noted that QUE suppressed the expression of the CLD-2 gene (Figure 4C). It is likely that QUE loosened TJ integrity through a mechanism other than the direct inhibition of ZO-1.

### 3.5. Pharmacological Investigation of the Mechanism of TJ-Opening Using HST or QUE

As mentioned above, not only the direct inhibition of the ZO-1(PDZ1)–CLD interaction, but also the other signaling pathways, are considered TJ-opening mechanisms using HST and QUE. In this context, we previously demonstrated that both BLI and BLE contributed to TJ-opening, partly through the ALK5-dependent pathway [18]. In addition, the inhibition of the MEK pathway signal partially reverted BLE-induced cell morphological changes [18]. Based on these considerations, we employed SB431542 or U0126 as ALK5 and MEK inhibitors, respectively, to determine whether they could revert the TJ-opening and the slender cell shape induced by either HST or QUE.

**Figure 5:**
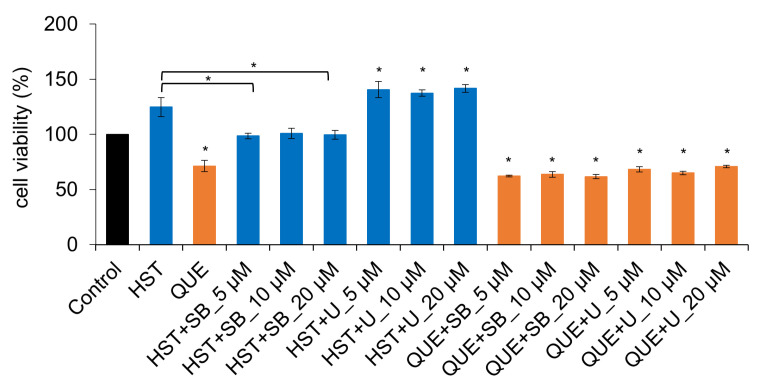
Comparison of cell viability in MDCK II cells exposed to flavonoids and inhibitors. Cells were treated with flavonoids at a concentration of 100 μM and/or SB431542 (labelled as SB)/U0126 5 (labelled as U), 10, and 20 µM for 48 h. Error bars indicate standard deviations. Statistical analyses were performed using Turkey-Kramer multiple comparison tests. These were different from the value of the control (DMSO) or HST-treated cells, * *p* < 0.05. HST, QUE: *n* = 4.

We first measured the number of viable cells using the WST-8 assay. The viable cells were 130% after treatment with HST and 60% after treatment with QUE compared to the control (Figure 5). When SB431542 was co-administered with HST, the enhanced cell proliferation reverted to the normal level. Therefore, HST is seemingly potent for the proliferation enhancement of MDCK II cells through the activation of the TGFβ pathway. To the contrary, either SB431542 or U0126 failed to revert the weak cytotoxicity using QUE, suggesting that the TGFβ and MEK pathways were not involved in QUE toxicity.

**Figure 6:**
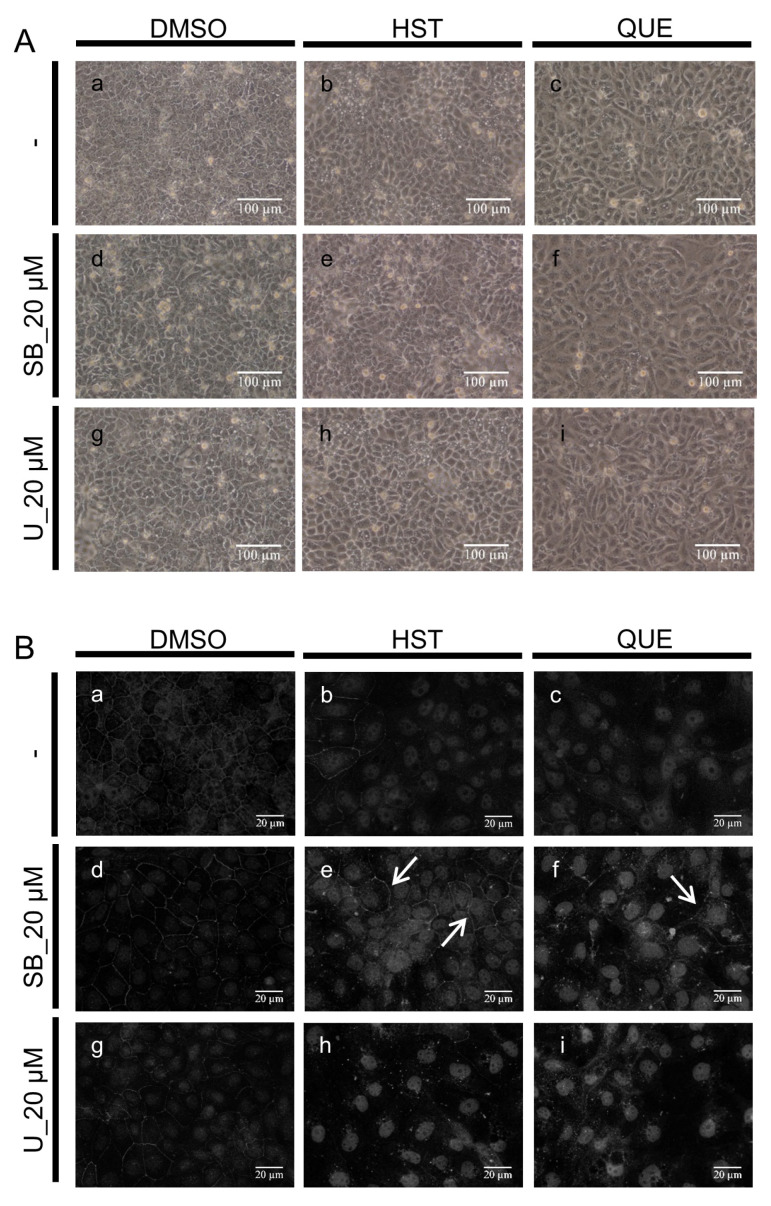
Effects of flavonoids on the morphology and TJ integrity of MDCK II cells. (**A**) DIC images with corresponding flavonoids are presented. Cells were exposed to flavonoids at a concentration of 100 μM and/or SB431542 or U0126 20 µM for 48 h. (**a**,**d**,**g**) control (DMSO); (**b**,**e**,**h**) HST; (**c**,**f**,**i**) QUE. Scale bar = 100 µM. (**B**) Immunofluorescence staining of CLD2 images are shown. Cells were treated with flavonoids at a concentration of 100 μM and/or SB431542/U0126 20 µM for 48 h. (**a**,**d**,**g**) control (DMSO); (**b**,**e**,**h**) HST; (**c**,**f**,**i**) QUE. Scale bar = 20 µM. Brightness is modified to 160%. Arrows indicate CLD-2 localized at partially restored tight junctions.

**Figure 7:**
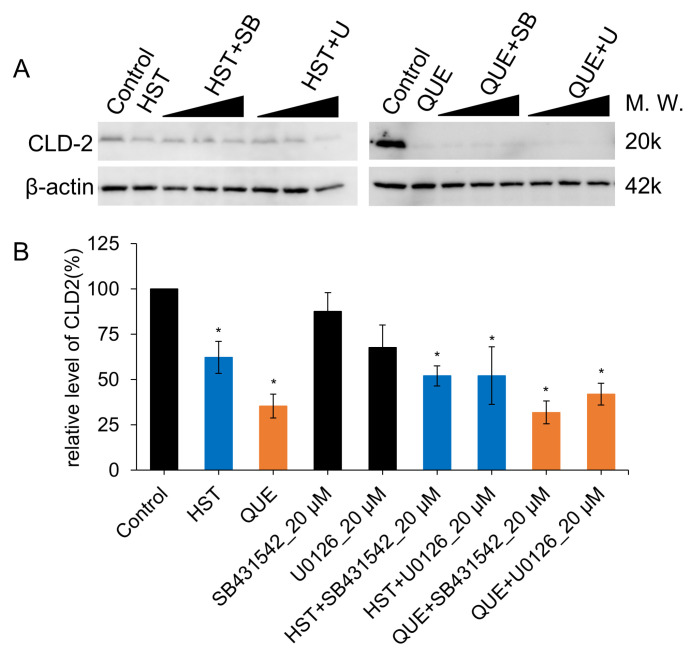
Changes in the relative amount of protein of CLD-2 after compounds treatment for 48 h in MDCK II cells. (**A**) Western blotting analysis of CLD-2 expressed in control (DMSO) and 100 µM flavonoid- and/or 5, 10, 20 µM SB431542/U0126 -treated MDCK II cells. (**B**) Quantitative analysis with densitometry of 100 µM flavonoid- and/or 5, 10, 20 µM SB431542/U0126- treated MDCK II cells. Turkey-Kramer multiple comparison tests were applied as statistical analyses. Error bars show standard deviations. Different from the value of the control cells, * *p* < 0.05. HST, QUE: *n* = 8.

We also examined the effects of SB431542 and U0126 on morphological changes in MDCK II cells under bright-field microscopy. The bright-field observation of viable cells showed that the co-exposure of HST with SB431542 resulted in the disappearance of white spots (Figure 6A panel e). This suggests that TGFβ/ALK5 could be involved in the appearance of white spots induced by HST. For the cell shape changes induced by QUE, either SB431542 or U0126 did not ameliorate the slenderized morphology of MDCK II cells (Figure 6A panel f,i). Thus, we ruled out that the QUE-induced slender cell shape was downstream of either the TGFβ or MEK pathway. Finally, we examined the recovery of the membrane localization and protein expression of CLD-2 using these inhibitors. SB431542 partially recovered the decreased localization of CLD-2 induced by HST (Figure 6B panel e). In addition, SB431542 could also weakly reverse the decreased CLD-2 by QUE at the TJ-area (Figure 6B panel f). However, in the Western blotting analysis, we did not observe a remarkable recovery of the CLD-2 amount through the co-administration of either SB431542 or U0126 (Figure 7A,B).

## 4. Discussion

HST is one of the major components of citrus flavonoids. It is found in the peels, fruits, and albedos of many citrus species. For example, fruits or fruit peels of *Citrus aurantium* L. (Rutaceae) contain HST, hesperidin (HSD), narirutin (NRT), neohesperidin (NHD), and kaempferol [34]. Conversely, several citrus fruits contain naringin (NAR) and naringenin (NRG), rather than HST-related flavonoids. In this study, we demonstrated a weak, but certain, TJ-opening activity of HST against MDCK II cells. However, we previously showed that NAR and NRG, but not NRT, had remarkable TJ-enhancing activity on MDCK II cells [21]. In the case of the NAR-related flavonoids, namely, NAR, NRG, and NRT, we previously showed that aglycone (NAR) exhibited stronger pharmacological effects than its glycosides. NRG, the neohesperidoside of NAR, showed mild TJ-enhancing activity, whereas NRT, the rutinoside of NAR, had no effect on TJ biogenesis. However, in the case of the glycosides of HST, including NHD and HSD, the neohesperidoside and the rutinoside of HST, respectively, showed TJ-enhancing activity, which is the opposing pharmacological action of TJ-opening induced by their aglycone HST [21]. It should be noted that some extracts from citrus peels or albedos are used as skin-conditioning ingredients for many cosmetics. However, the active ingredients of these citrus extracts must contain both TJ-enhancing and TJ-opening flavonoids. Therefore, when using citrus extracts as an additive for cosmetics for skin protection purposes, the extraction method of citrus may become particularly important. In this context, further studies on the quantitative comparison of the pharmacological effects of the TJ-opening activity of HST in simultaneous administration of other TJ-enhancing flavonoids, such as NAR and NRG, are needed.

Accordingly, some studies have reported the pharmacological activities of HST independent to its antioxidant activity, such as hypolipidemic (cholesterol-lowering) activity, anti-cancer activity, anti-metastatic activity, and anti-aromatase activity [34,35,36,37]. According to this hypolipidemic activity, HST has been reported to increase the expression of low-density lipid receptor gene probably via sterol regulatory element (SRE)-binding proteins [35]. HST has also been reported to reduce the expression of genes encoding acyl-coenzyme A: cholesterol acyltransferase (ACAT1 and ACAT2) toward lowering cholesterol levels [38]. Another beneficial effect of HST includes activation of Nrf2 signaling pathway in several cells [36,37,38]. HST may have antioxidant, anti-inflammatory, anti-allergic, and vaso-protective actions [20]. HST also inhibits snake venom protease [39], as well as some virus genome-derived proteases, including zika, chikungunya, and dengue viruses [40,41,42]. HSP was previously suggested as a template molecule to develop new anti-arrhythmic drugs, as it blocks slowly-inactivating currents carried by the type 3 long QT syndromes (LQT3)-associated voltage-gated Na^+^ channel (hNaV1.5) channel mutant R1623Q, an arrhythmogenic gain-of-function mutant of hNaV1.5 [43]. Finally, HST is expected to become a potential neuroprotective prophylactic (reviewed in [44]). None of these pieces of pharmacological evidence foreshadowed HST’s TJ-opening activity shown in this study.

**Figure 8:**
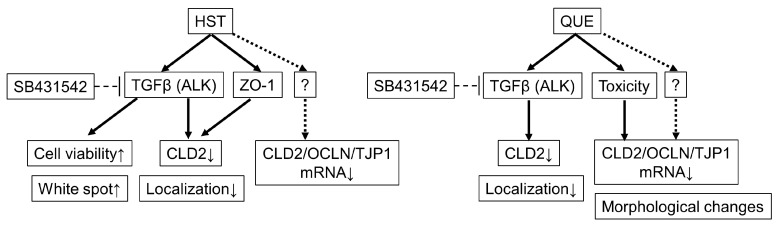
Putative molecular mechanisms underlying TJ modulation induced by HST (**left panel**) and QUE (**right panel**). Solid arrows indicate either stimulation or enhancement, dashed arrows indicate the possibility of stimulation, and dashed long left tacks indicate inhibition. “?” represents an unknown factor. ↑: increase; ↓: decrease.

Therefore, we systematically examined how a high-dose treatment of both HST and QUE against epithelial cells had a suppressive effect on the subcellular localization of CLD-2 in the TJ compartment of the lateral membrane. Note that the TJ-opening activity of flavonoids is thought to be independent from their antioxidative property. In general, oxidative stress has a detrimental effect against TJ-integrity [45,46]. Accordingly, close examination revealed that the pharmacological activity of HST to suppress the protein level of CLD-2 was weaker than that of QUE. Similarly, HST was milder than QUE in suppressing the mRNA levels of TJ-related genes, including *CLD-2*, *OCLN*, and *TJP1* (ZO-1). One of the differences in the biological mechanisms between HST and QUE is the presence or absence of a direct interaction between the flavonoids and ZO-1(PDZ1), which is the responsible interface of ZO-1 to the C-terminal PDZ-binding motifs of CLDs [7]. Although the physiologically relevant target of QUE in MDCK II cells is still unclear, we demonstrated that QUE revealed a strong suppression of the expression of the three TJ-related genes. We also showed that both HST and QUE partly activated the TGFβ signaling pathway in a different manner. We concluded that the pharmacological actions of HST and QUE in the proliferation and TJ opening of MDCK II cells were only partly dependent on the TGFβ/ALK5 pathway, regardless of whether the other unknown signaling pathway exists, especially for regulating the expression of TJ-related genes. Figure 8 summarizes the pathways affected by the HST and QUE examined in this study. Table 1 presents the pharmacological effects of the four flavonoids: HST, QUE, BLI, and BLE. As BLI and BLE are also known to have TJ-opening activities by stimulating the TGFβ pathway [18], the pharmacological action of HST resembles that of BLI, rather than QUE, because HST did not induce the morphological changes. However, according to the subcellular localization of TJ-components, HST and QUE are distinct from BLI and BLE because the former decreased OCLN and ZO-1 level. We further assessed these pharmacological activities against some antioxidant capacity parameters, such as Trolox equivalent antioxidant capacity (TEAC), ferric reducing antioxidant power (FRAP), 2,2-diphenyl-1-picrylhydrazyl (DPPH) assay, and Folin-Ciocalteu reducing capacity (FCR) from the literature (Table 1) [47,48]. All these parameters suggested that Que (and BLE) are stronger antioxidants than HST. Contrary to our expectations, we found a weak positive correlation between typical antioxidant capacities and TJ-opening activity. In general, oxidative stress is thought to be harmful against TJ integrity [49]. Finally, it should be noted that HST, BLI, and BLE showed weak, but certain, direct interaction to the canonical ligand binding pocket of ZO-1(PDZ1). Currently, however, critical functional groups of flavonoids for PDZ-binding remain unclear. Not only the number and the position of hydroxyl groups, but also the molecular shape, may be important for ZO-1(PDZ1) interaction, as suggested by our HADDOCK study (Supplementary Figure S5). Recently, BLI has been reported as an efficient absorption enhancer of oral administration of insulin to rats when combined with AlCl_3_ nanoparticles [50]. Conversely, QUE and BLE exhibited the activity of morphological changes in the epithelial cell shapes, which become concerning in relation to the adverse effects of these flavonoids. Therefore, HST is considered as another flavonoid candidate for naturally occurring epithelial drug absorption enhancers, such like BLI.

## 5. Conclusions

This study demonstrated that HST and QUE downregulated the subcellular localization of CLD-2 in the TJ compartment of the lateral membrane. HST promoted the proliferation of MDCK II cells, partly through the TGFβ pathway, whereas QUE suppressed the cells. QUE, but not HST, induced morphological changes in MDCK II into a slenderer cell shape. QUE, but not HST, attenuated the gene expression of TJ-related genes, including CLD-2, occludin, and ZO-1. Only HST, but not QUE, was directly bound to ZO-1(PDZ1), which was the responsible interface between ZO-1 and CLDs’ C-terminal. These results suggest that HST is an attractive candidate for developing a naturally occurring drug absorption enhancer for the paracellular route. 

## Figures and Tables

**Table 1:** Comparison of pharmacological effects of flavonoids against TJ of MDCK II cells.

	HST	QUE	BLI ^a^	BLE ^a^
CLD-2				
TJ localization	− −	− − −	− − −	− − −
mRNA expression	−	− − −	−	−
OCLN				
TJ localization	−	−	+/−	+/−
mRNA expression	−	− − −	n.ex.	n.ex.
ZO-1 expression				
TJ localization	−	−	+/−	+/−
mRNA expression	−	− − −	n.ex.	n.ex.
MDCK II cell				
Proliferation	+ +	− −	+/−	−
Slenderer cell shape	no	yes	no	yes
Direct ZO1(PDZ1) interaction	yes	no	yes	yes
Other signaling pathways				
TGFβ	partially	partially	yes	yes
MEK/ERK	partially	no	partially	yes
Antioxidant capacity from literature				
TEAC (trolox equiv./mmol) ^b^	2.01 ± 0.04	5.72 ± 0.16		
FCR (chlorogenic acid equiv./mmol ^b^	0.53 ± 0.03	1.24 ± 0.09		
DPPH (trolox equiv./mmol) ^b^	0.48 ± 0.04	2.25 ± 0.09		
FRAP (ferrous equiv./μmol) ^c^	21.1 ± 0.4	95.9 ± 5.4		38.9 ± 2.5

^a^ Hisada et al., 2020 [18]; ^b^ Zhang et al., 2011 [47]; ^c^ Firuzi et al., 2005 [48]; n.ex. Not examined. Symbols +, −, and +/− indicates increase, decrease, and un-changed, respectively. The number of the symbols reflects degree of change.

## Data Availability

The data are contained within this article and Supplementary Materials.

## Supplementary Materials

The following supporting information can be downloaded at: https://www.mdpi.com/article/10.3390/antiox12040952/s1, Figure S1: Effects of quercetin (QUE) on cell morphology in MDCK II cells; Figure S2: Close-up view of HST-treated cells. Effects of HST on the morphology of MDCK II cells (enlarged one); Figure S3: Effects of flavonoids on TJ integrity of MDCK II cells (original figures); Figure S4: Effects of flavonoids on the shape and TJ integrity of MDCK II cells (original figures); Figure S5: Direct interaction between ZO-1(PDZ1) and the flavonoids; Figure S6: Effects of flavonoids on the morphology and TJ integrity of MDCK II cells (original figures); Figure S7: 600MHz ^1^H NMR spectra of the commercially purchased reagents used in study.
