# Supplementary material for: Pharmacologic Comparison of High-Dose Hesperetin and Quercetin on MDCK II Cell Viability, Tight Junction Integrity, and Cell Shape
Source: Antioxidants (Basel). 2023 Apr 18;12(4):952. doi: 10.3390/antiox12040952 (PMC10135814; doi:10.3390/antiox12040952)
Supplement: Supplementary file 1 [file antioxidants-12-00952-s001.zip › antioxidants-2249147-supplementary.pdf]

SUPPLEMENTARY INFORMATION

Pharmacologic Comparison of High-Dose Hesperetin and Quercetin on MDCK II Cell Viability, Tight Junction Integrity, and Cell Shape

Mio Nakashima [1], Natsuko Goda [1], Takeshi Tenno [1,2], Ayaka Kotake [3], Yuko Inotsume [3], Minako Amaya [3], and Hidekazu Hiroaki [1,2,4,*]

[1] Laboratory of Structural Molecular Pharmacology, Graduate School of Pharmaceutical Sciences, Nagoya University, Furocho, Chikusa-ku, Nagoya, Aichi, 464-8601, Japan
nakashima.mio.nagoya.u@gmail.com (M.N.),
tenno.natsuko.b0@f.mail.nagoya-u.ac.jp (N.G.),
tenno.takeshi.k6@f.mail.nagoya-u.ac.jp (T.T.),
hiroaki.hidekazu.j7@f.mail.nagoya-u.ac.jp (H.H.)

[2] BeCerllBar, LLC, Business Incubation Building, Nagoya University, Furocho, Chikusa ku, Nagoya, Aichi 464 8601, Japan

[3] Cosmetics Research Department, Nicca Chemical Co. Ltd., Fukui, Fukui, 910-8670, Japan
a-kotake@demicosmetics.com (A.K.),
y-inotsume@demicosmetics.com (Y.I.),
m-amaya@demicosmetics.com (M.A.)

[4] Center for One Medicine Innovative Translational Research, Gifu University Institute for Advanced Study, Yanagito, Gifu, Gifu, 501-1112, Japan

* Correspondence: hiroaki.hidekazu.j7@f.mail.nagoya-u.ac.jp;
Tel.: +81-52-789-4535

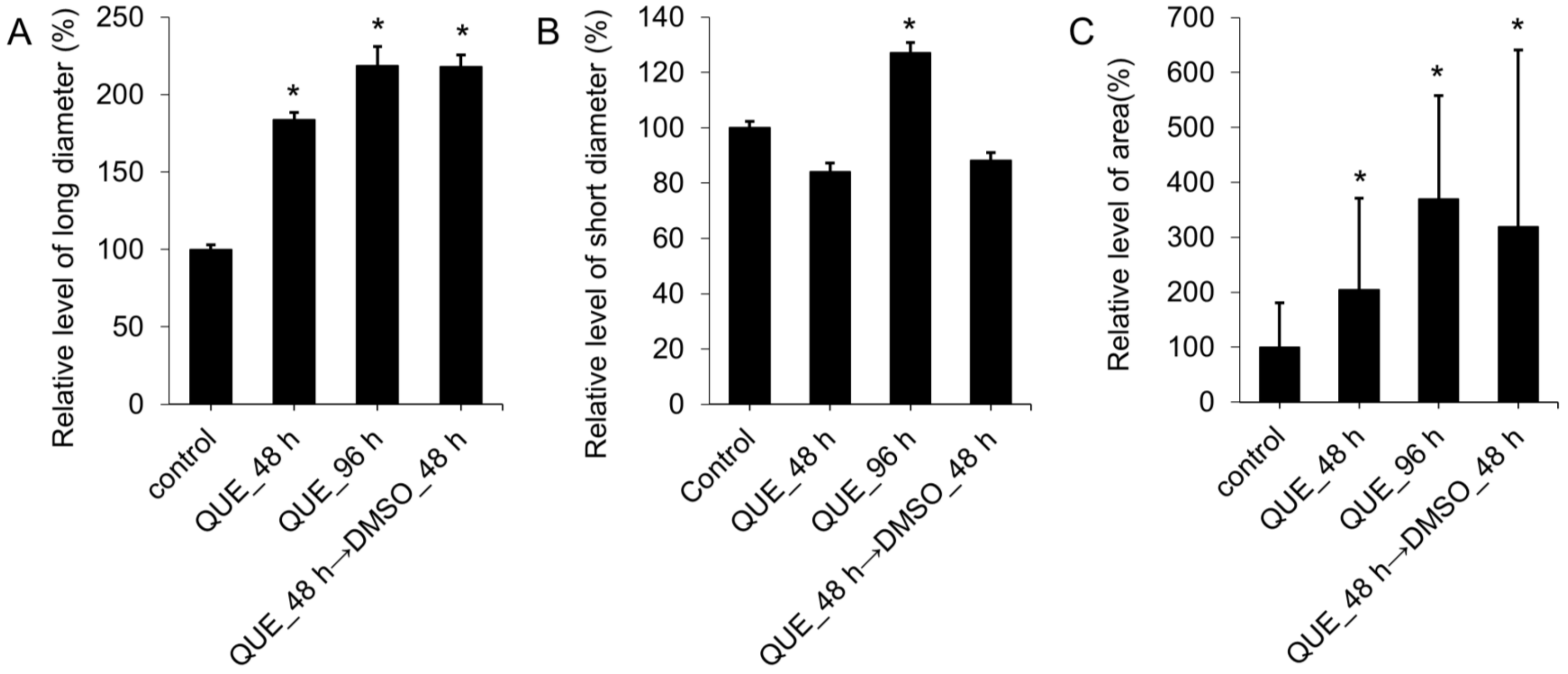

Supplementary Figure S1

Effects of quercetin (QUE) on cell morphology in MDCK II cells. Statistical analyses were performed by Turkey-Kramer multiple comparison tests. Relative level of long diameter, (B) short diameter and (C) area. Different from the value of the control cells, $p<0.05$. n=11. MDCK: Madin-Darby canine kidney, DMSO: dimethylsulfoxide, HST: hesperetin, QUE: quercetin.

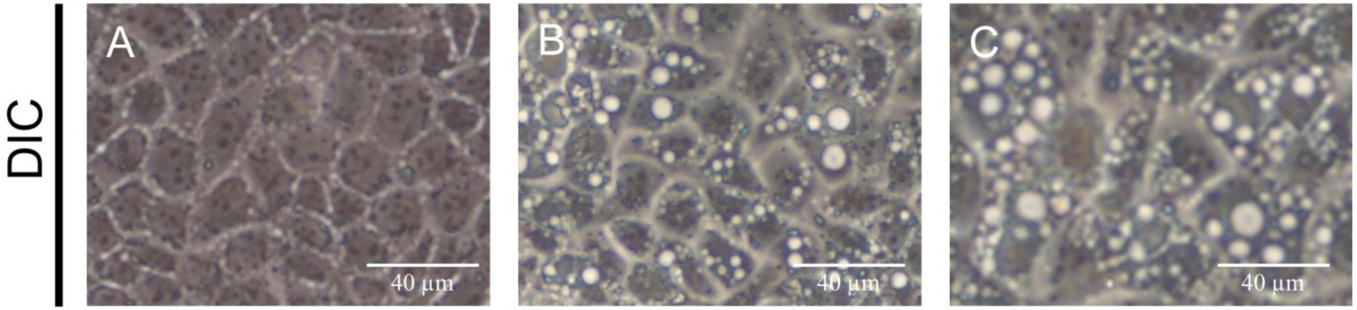

Supplementary Figure S2

Close-up view of HST-treated cells. Effects of HST on the morphology of MDCK II cells (enlarged one). Bright-field differential interference contrasts (DIC) the images with corresponding flavonoids that are arrayed. Cells were treated with HST at a concentration of 100 µM for 48 hours. Scale bar = 40 µm. control (DMSO), (B), (C) HST.

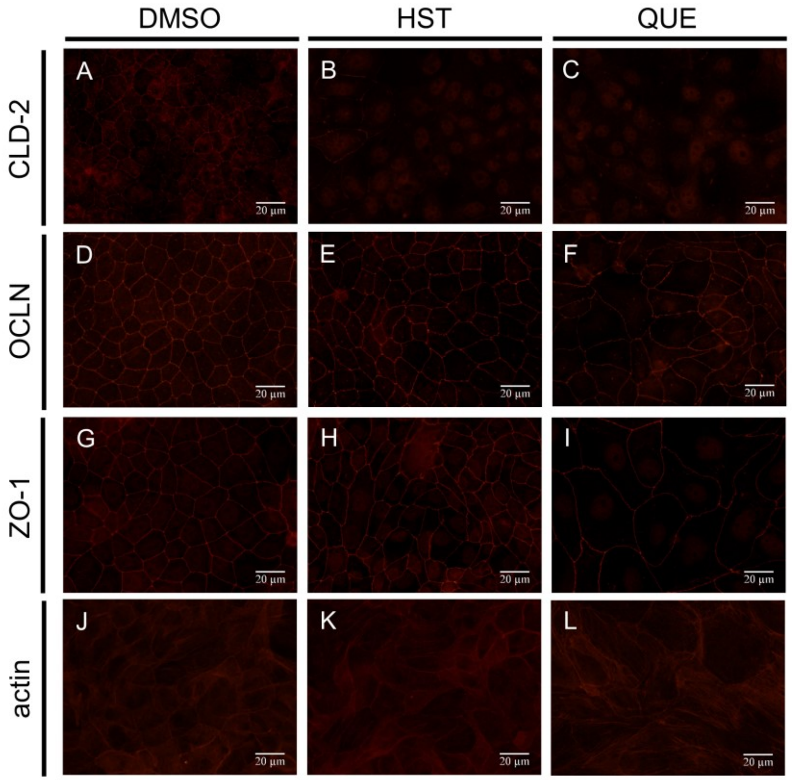

Supplementary Figure S3

Effects of flavonoids on TJ integrity of MDCK II cells (original figures). Immunofluorescence staining of CLD-2, ZO-1, OCLN, actin images are arrayed. Cells were treated with flavonoids at a concentration of 100 μM for 48 hours. (A), (D), (G), (J) control (DMSO); (B), (E), (H), (K) HST; (C), (F), (I), (L) QUE. Scale bar = 20 μM.

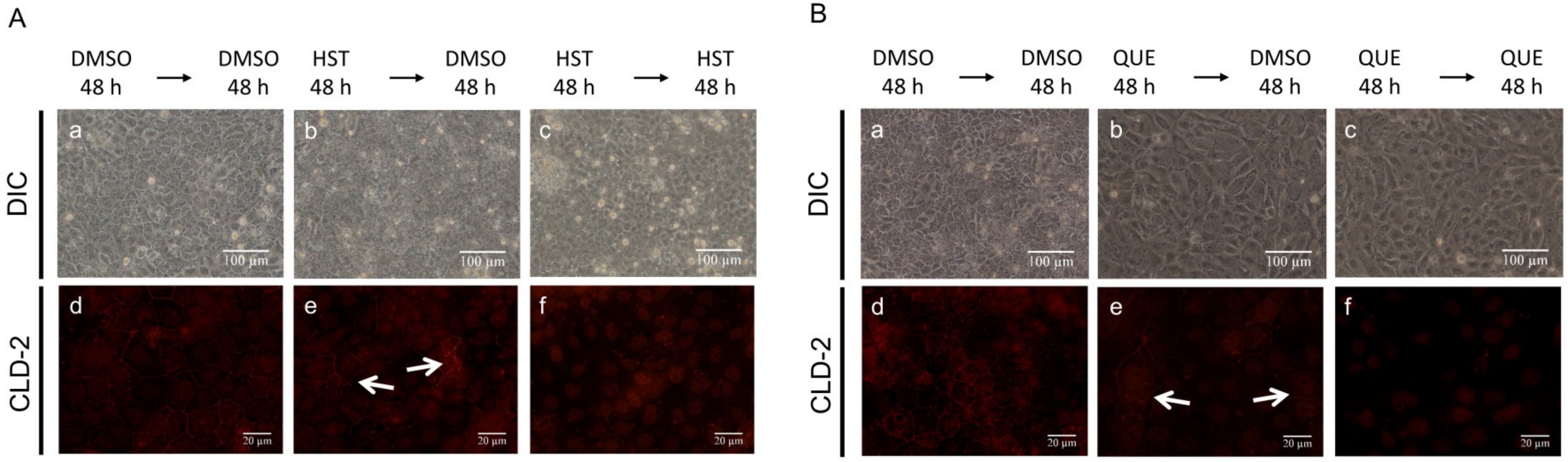

Supplementary Figure S4

Effects of flavonoids on the shape and TJ integrity of MDCK II cells (original figures). Bright-field differential interference contrast (DIC) images (a-c) and immunofluorescence staining of CLD-2 images (d-f) of MDCK II cells are arrayed. Cells were treated with HST or QUE at a concentration of 100 µM for 48 hours and then treated with HST, QUE or DMSO at a concentration of 100 µM for 48 hours after washed with medium. (a, d) control (DMSO-DMSO), (b, e) flavonoid-DMSO and (c, f) flavonoid-flavonoid. Scale bar = 100 µm (a-c), 20 µm (d-f).

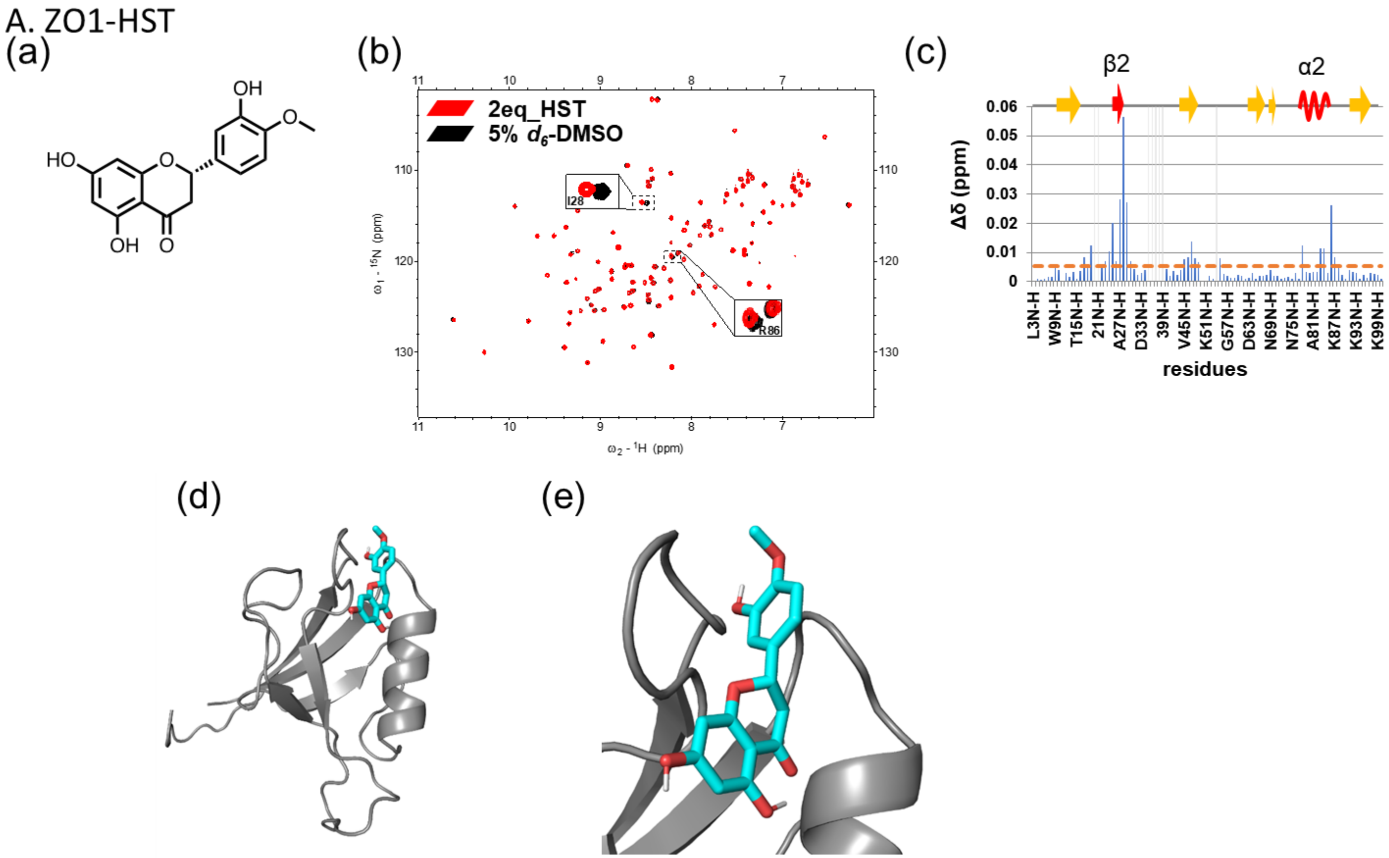

Supplementary Figure S5

B. ZO1-QUE

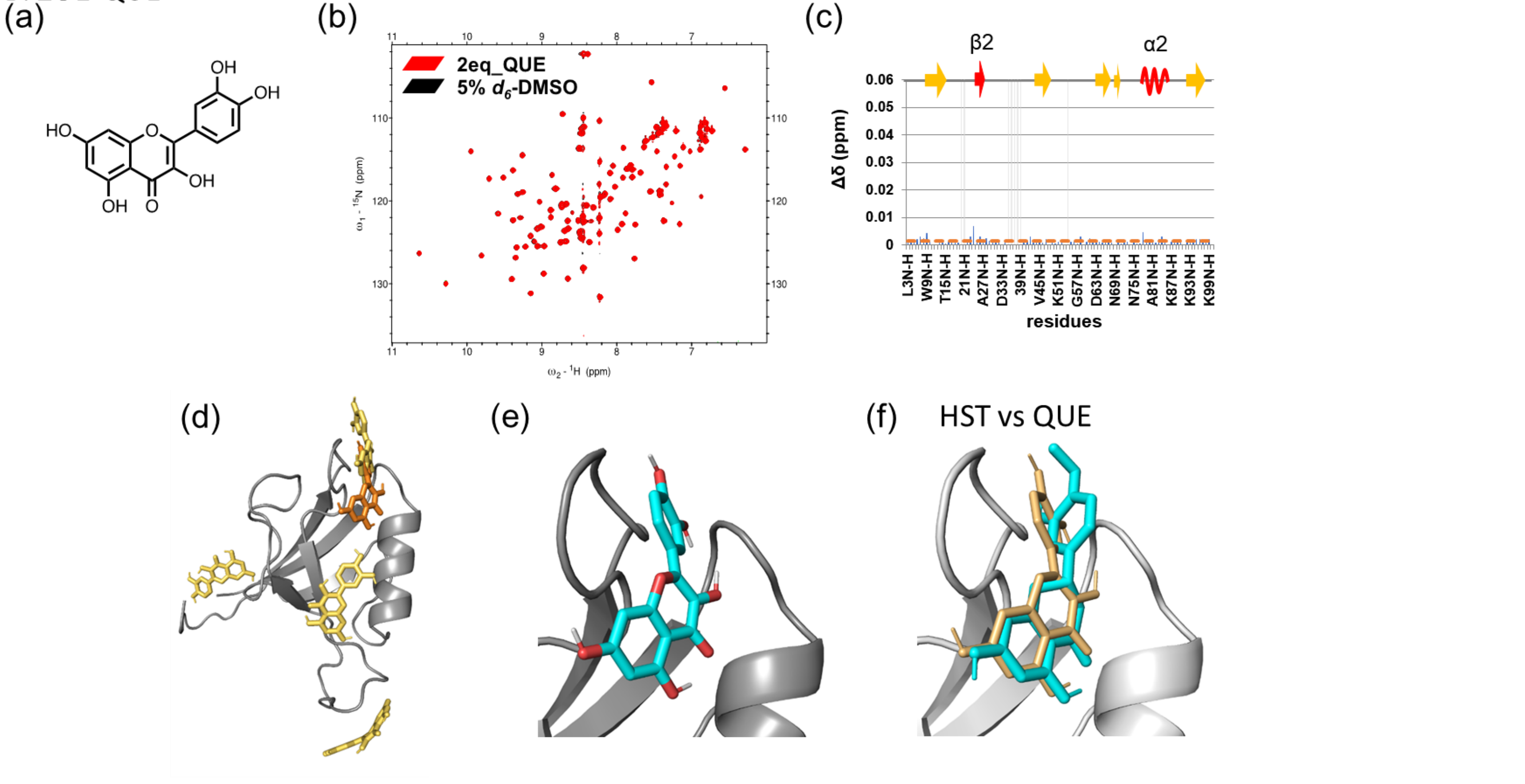

Supplementary Figure S5 (continued)

Direct interaction between ZO-1(PDZ1) and the flavonoids. (a) Chemical structure of the flavonoids titrated. (b) Overlaid HSQC spectra of 0.1 mM ZO-1(PDZ1) in the absence (black) and presence of 2 equivalent (red) of HST (A) and QUE (B) respectively. (c) Normalized chemical shift changes in the presence of 2 equivalent of HST (A) and QUE (B). (d) Best one or best five poses of the ZO1(PDZ1) with HST (A) or QUE (B). (e) Close-up view of the best pose of the ZO1(PDZ1) with HST (A) or QUE (B). (f) Overlay of HST (cyan) and QUE (gold) docked to ZO1(PDZ1). DMSO: dimethylsulfoxide, HST: hesperetin, QUE: quercetin.

Method section for Supplementary Figure S5

Molecular docking based on NMR chemical shift perturbations.

Structural models of the ZO1-HST and ZO1-QUE complexes were calculated with the NMR structure of ZO-1(PDZ1) (PDB ID of 2RRM) [16] using HADDOCK software (HADDOCK2.4) [27,28]. We used the first model registered in 2RRM for the docking experiment. Before starting the docking experiments, N- and C-terminal residues were charged. The coordinates of the flavonoids were obtained from the webserver (https://molview.org) and converted into pdb format. The data of CSPs were used as the restraints to generate the NMR-based docking model according to the user manual. Thus, the residues of ZO-1(PDZ1) that showed marked CSPs (average + 1σ) were defined as the binding sites of ZO-1(PDZ1). The residues A12, F17, G18, A20, I21, S22, S40, H71, Q76, Q77, and R79 were set as "active residues", and the passive residues were defined automatically. For the ZO1-QUE docking model, no active residues were predefined. Instead, "random patch" option was set to "on". The other parameters for HADDOCK experiment were default. The ZO1-HST structure with the lowest Z score and the ZO1-QUE structures with the five lowest Z score were selected and displayed using PyMOL.

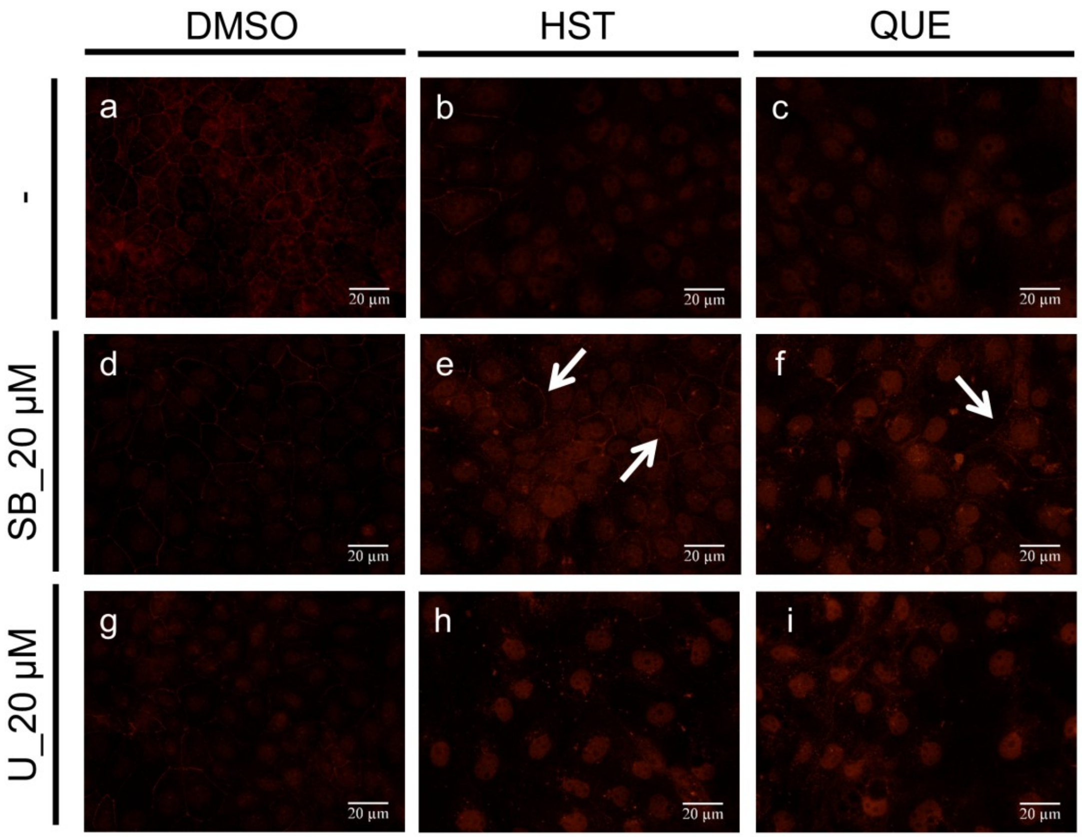

Supplementary Figure S6

Effects of flavonoids on the morphology and TJ integrity of MDCK II cells (original figures). Immunofluorescence staining of CLD2 images are arrayed. Cells were treated with flavonoids at a concentration of 100 μM and / or SB431542 / U0126 20 μM for 48 hours. (a), (d), (g) control (DMSO); (b), (e), (h) HST; (c), (f), (i) QUE. Scale bar = 20 μM.

A. Hesperetin (HST)

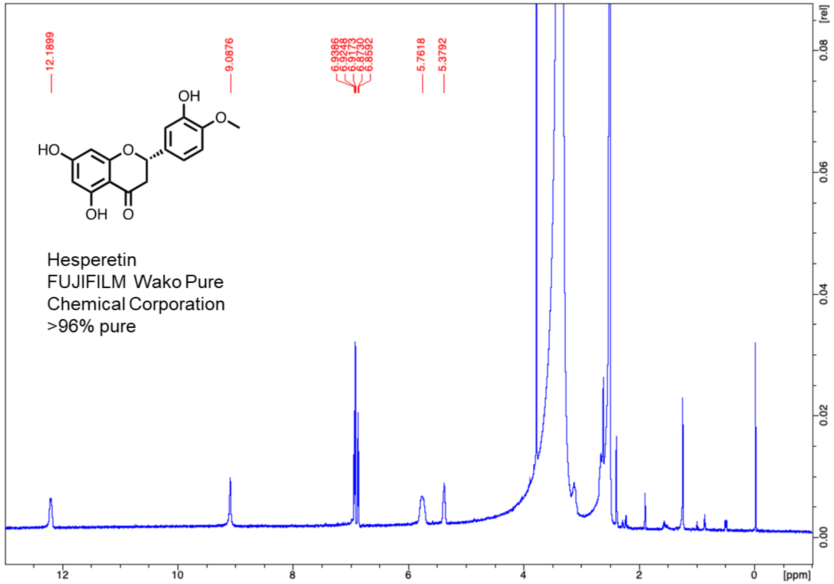

B. Quercetin (QUE)

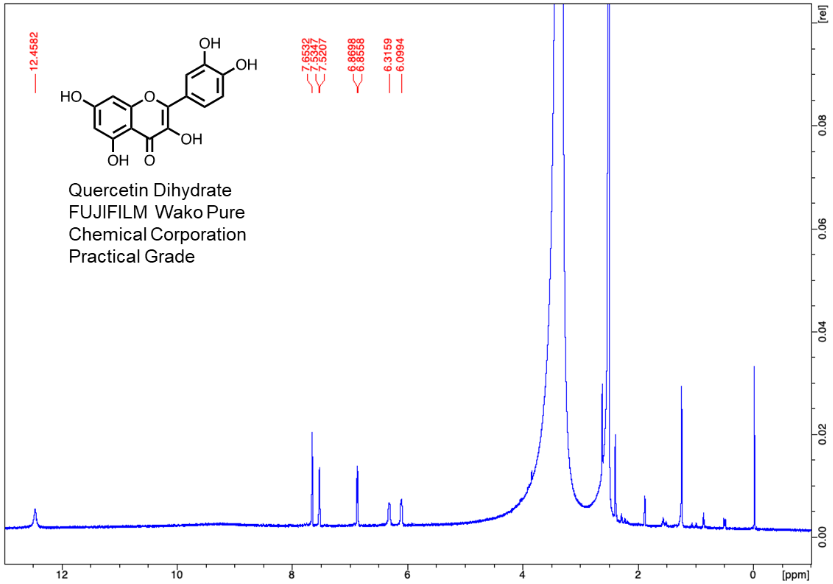

C. DMSO-*d6*

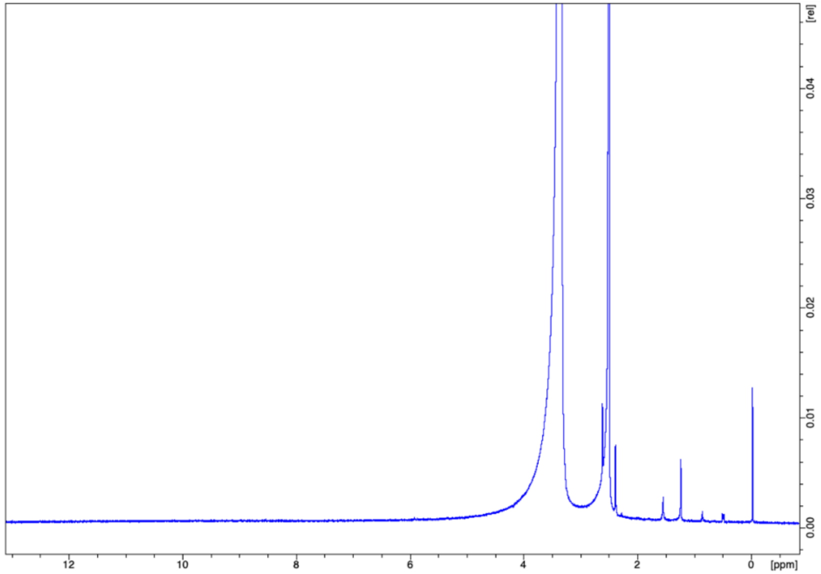

Supplementary Figure S7

600MHz ^{1}H NMR spectra of the commercially purchased reagents used in study. (a) hesperetin (HST), (b) quercetin (QUE), (c) dimethyl sulfoxide (DMSO-d_6). All ^{1}H-NMR spectra were measured in DMSO-d_6 and sodium trimethylsilyl-propanesulfonate (DSS) as internal standard. Some peaks of solvent and residual water and chemicals were observed (DMSO-d_6: 2.5 ppm, n-hexane: 0.87, 1.25 ppm, water 3.36 ppm).
